# Clinical and Environmental Predictors of Health-Related Quality of Life in Lithuanian Children and Adolescents with Spina Bifida: A Cross-Sectional Analysis of a Nationally Represented Sample

**DOI:** 10.3390/medicina54040059

**Published:** 2018-08-28

**Authors:** Indrė Bakanienė, Audronė Prasauskienė

**Affiliations:** Department of Children’s Rehabilitation, Lithuanian University of Health Sciences, LT-4717910 Kaunas, Lithuania; a.prasauskiene@gmail.com

**Keywords:** children, environment, spina bifida, quality of life

## Abstract

*Background and objectives*: In pediatric chronic health conditions, health-related quality of life (HRQOL) is a useful indicator of health, development, and well-being. The purpose of the study was to assess the effect of clinical and environmental factors on the HRQOL of children and adolescents with spina bifida (SB). *Materials and methods*: A cross-sectional study of the sample of 99 children and adolescents with SB aged 5 to 17 years. The questionnaires used in the study were the Spina Bifida Health-Related Quality of Life instrument (HRQOL-SB), and the Participation and Environment Measure for Children and Youth. Medical data were obtained from the medical records and the clinical examination. *Results*: A multivariate linear regression revealed that the most potent predictors of the HRQOL in children with SB were the community overall environmental supports (β = 0.504; *p* = 0.0001), a number of health conditions (β = −0.395; *p* = 0.0001), access to personal transportation (β = 0.236; *p* = 0.023), and supplies (β = 0.181; *p* = 0.031), explaining 80.3% of the variance in the SB-HRQOL scores. The most significant predictors of the HRQOL in adolescents were a number of health conditions (β = −0.387; *p* = 0.0001), cognitive demands of activities at home (β = 0.345; *p* = 0.0001), supplies (β = 0.267; *p* = 0.0001), money (β = 0.303; *p* = 0.0001), physical layout at school (β = 0.188; *p* = 0.008), and access to public transportation (β = 0.206; *p* = 0.019), explaining 89.5% of the variance in the SB-HRQOL scores. *Conclusions*: Both clinical and environmental factors determined the HRQOL of children and adolescents with SB. Environmental supports and resources contributed to HRQOL more than medical problems, especially in adolescents. The number of associated medical problems, reflecting disease severity, was the more potent clinical predictor compared to an individual health problem.

## 1. Introduction

Spina bifida (SB) is the most complex congenital disability compatible with long-term survival. Children with SB typically have multiple medical problems (e.g., paralysis and sensory dysfunction, orthopaedic abnormalities, urinary and bowel incontinence) with the severity related to the type of malformation, the level of lesion, and the extent of the brain and the spinal cord injuries [1,2]. Limitations of activity limitations and restrictions of participation caused by the physical and mental disabilities as well as a multitude of comorbidities in children with SB may negatively affect their health-related quality of life (HRQOL) [2].

HRQOL is a multi-dimensional concept, which measures an individual’s perception of their physical, psychosocial, and role functioning concerning their health [3]. An important aspect of the HRQOL concept is how the manifestation of an illness or treatment is experienced by an individual. For children with chronic health conditions, the goal of healthcare is to assure the fullest health possible by improving symptom management and their ability to cope with the negative impact of their condition. For this reason, some researchers have indicated that measuring of HRQOL may be more important than examining biomedical parameters to evaluate the effect of chronic disease and the impact of medical interventions [4].

The more recent review by Bakaniene et al. (2016) has identified 43 papers on the HRQOL of children and adolescents with SB. All the studies (except one) were focused on nonmodifiable demographic and illness-specific correlates, while the impact of modifiable environmental factors (which might be more important to practitioners and policymakers) was not investigated. Moreover, most of the researchers investigated the potential clinical determinants separately and got controversial results regarding the impact of pathology (e.g., level of lesion paralysis, incontinence) as well as different treatment strategies on the HRQOL [2]. The complexity of SB can account for such findings: children with SB have multisystem physiological and functional impairments, so less severe disorder or improvement in just one affected system may not be sufficient to improve patient’s overall perceptions of HRQOL [2,5].

The aims of our study were to: (1) investigate the collective effects of health conditions and functional impairments on the HRQOL of children and adolescents with SB; (2) analyse the impact of environmental variables on the HRQOL. We hypothesized that a higher number of health conditions and functional issues would be the more potent predictor of the HRQOL than separate health problems or functional limitation. We also hypothesized that a higher number of environmental support and resources, as well as a lower number of environmental barriers, would be associated with better HRQOL.

## 2. Methods

A cross-sectional study of a nationally representative sample of children with SB was conducted from May to October 2017. This was a part of the extensive national survey on the functioning of children with SB. Ethical approval was received from Kaunas Regional Biomedical Research Ethics Committee (May 28, 2016; No. BE-2-37).

Data of children with SB were obtained from the medical files of the hospital of Lithuanian University of Health Sciences, which is the only hospital in Lithuania where the SB closure surgery is performed. All Lithuanian children with SB undergo the SB closure operation; therefore, we were able to find the data of all Lithuanian children with SB that were born alive.

### 2.1. Sample

The study group consisted of children with SB born between 1999 and 2012, living in Lithuania at the time of the study. Eligible children and their parents were contacted both by a letter of invitation or by a personal request from the study and clinical staff during a clinic visit. Inclusion criteria for this study were the following: (a) age of 5 to 17 years, (b) no major medical condition (i.e., life-threatening, progressive, or incapacitating disability) other than SB.

### 2.2. Procedure

All 112 families that have children with SB were contacted by the study staff and further informed. The written informed consent from both the parent and children older than 12 years of age was obtained. The structured interviews and clinical examination took place at home (36%) or in a hospital setting (64%). Also, the HRQOL questionnaire was subsequently mailed to 20 children and 20 parents after three weeks to complete the questionnaire again to check test–retest reliability. Information on hydrocephalus, shunt surgery, dislocation of the hip(s), spinal deformities, radiographs, and intelligence quotient was obtained from the medical records.

### 2.3. Measurements

#### 2.3.1. Health-Related Quality of Life

Perception of HRQOL was assessed using the Spina Bifida Health-Related Quality of Life questionnaire (SB-HRQOL), developed by Parkin et al. (1997). This questionnaire is the most widely used SB specific instrument to assess HRQOL in children and adolescents [2]. It is formed of two scales: 44 questions for the 5–12-year age group and 47 questions for the 13–20-year age group. Items are scored on a 5-point Likert scale, and the final score is obtained by summing positive items and subtracting negative ones. The scores range from 44 to 220 for children and 47 to 235 for adolescents [6].

For children, the questionnaire was used as a proxy report, whereas adolescents completed the questionnaire themselves. Also, adolescents responded to the global question “How would you rate your quality of life?” and parents of children 5–12 years responded to the question “How would you rate your child's quality of life?” Both overall QOL items were reported on a scale of 0 to 100.

In a reference group of Canadian children with SB, reproducibility of the SB-HRQOL was good (Intraclass correlation coefficient, ICC = 0.78 for children; ICC = 0.96 for adolescents), and internal consistency was good (Cronbach’s alpha α = 0.93 for children; α = 0.94 for adolescents). Also, validity, measured by relating the instrument to a global question of a child’s well-being, was very good in both age groups (r = 0.57 for children; r = 0.63 for adolescents) [6].

The translation into Lithuanian of the SB-HRQOL was performed according to guidelines described by Wild et al. (2005) [7]. The original SB-HRQOL version was independently translated by two native Lithuanian speakers with excellent knowledge of English. A third independent translator, also a native Lithuanian speaker, reconciled the forward translations by creating a hybrid version. The reconciled version was then back-translated by a native English-speaking translator who was fluent in Lithuanian. The back-translated and original versions of the questionnaire were compared and the results were used for the development of the final consensus Lithuanian version of the SB-HRQOL.

Reliability and validity of the translated versions of the SB-HRQOL were calculated from the study sample. Cronbach’s alpha in this study sample was good (0.93 for children; 0.85 for adolescents). Test–retest reliability was good (ICC = 0.77 for children; ICC = 0.77 for adolescent). Validity, measured by relating the instrument to a global question of child’s well-being, was very good for children (r = 0.73), and acceptable for adolescents (r = 0.49).

#### 2.3.2. Clinical Factors Associated with Health-Related Quality of Life

The condition variables included a level of lesion, ambulation, hydrocephalus, mental abilities, orthopedic deformities, urinary and bowel incontinence. Determination of the lesion level was based on muscle strength of the lower extremities using a four-item categorization [8]: (1) thoracic—the lack of volitional leg movements; (2) high-lumbar (L1–L3)—absent or less than fair strength of the quadriceps and musculature below on functional muscle testing; (3) low-lumbar (L3–L5)—normal quadriceps strength and no plantar flexor strength; (4) sacral—active plantar flexion.

Ambulatory status was classified using the modified Hoffer’s classification (2005): community ambulatory, household ambulatory, therapeutic ambulatory, and nonambulatory [9]. Joint flexion contractures were defined for the ankle, knee, and hip. The hip was defined as dislocated with a Reamer index of 100%. Spine deformity was defined as either scoliosis with Cobb angle > 30 and/or kyphosis > 70 or a previously surgically treated spine deformity with no curvature at the present examination.

Bladder continence was defined as being dry during the day, and bowel continence was defined as no involuntary leakage during the day. Incontinence was coded as “1” for children who used incontinence pads or diapers only sometimes for safety reasons and as “2” for those who used diapers permanently.

Information on mental status, such as Intelligence Quotients (IQ) (Wechsler intelligence Scale for Children, third edition, WISC-III) was obtained from the medical records. IQ < 70 was considered as an intellectual disability: mild (IQ 50–69), moderate (IQ 35–49), severe (IQ 20–34), and profound (IQ < 20). Hydrocephalus status was coded 0 if none and one if shunted and then the numbers of revisions were added. A mean number of health problems and functional issues were calculated to represent the complexity of the condition with each possible health problem or functional limitation being counted as “1”. The same coding system was applied in the studies of Anaby et al. (2013) [10] and Marino et al. (2018) [11].

#### 2.3.3. Environmental Factors Associated with Health-Related Quality of Life

Environmental variables were assessed using the Participation and Environment Measure for Children and Youth (PEM-CY). The PEM-CY is a parent-reported instrument that examines the participation and environment of children and youth ages 5–17 years across three settings: home, school, and community. For each setting, environmental aspects that affect the child’s participation (e.g., attitudes, accessibility, resources, and availability of programs) are estimated separately: 13 environment-related items for the home, 17 for school, and 16 for the community. These items capture environmental resources (access to transportation, information, services, equipment or supplies, time, and money) and supports (physical layout, sensory qualities, physical, cognitive, and social demands, child’s relationship with others, and attitudes and actions of surrounding people). Parents are asked whether each item helps or makes it harder for their child to participate in activities in that setting (not an issue, usually helps, sometimes helps/sometimes makes harder, usually makes harder) or about perceived adequacy/availability of resources (not needed, usually yes, sometimes yes/sometimes no, usually no). PEM-CY summary scores that were calculated in the study are provided in Table 1. Prior research indicated that the PEM-CY is reliable (internal consistency 0.59–0.91; test–retest reliability 0.58–0.95) and able to distinguish the participation and perceived supportiveness of the environment between children with and without disabilities [12].

The translation into Lithuanian of the PEM-CY was performed in the same way as the SB-HRQOL translation. Cronbach’s alpha for the environment scales was 0.49, 0.71, and 0.72 for the supportiveness, and 0.79, 0.72, and 0.76 for the resources at home, school, and the community, respectively. These estimates are acceptable and similar to the English version of the PEM-CY except for the internal consistency of the home supportiveness scale. Construct validity was assessed by hypothesis testing whether a desire for change scores was associated with the environmental supportiveness total score. There was a significant negative correlation between parents’ desire for change in their child’s participation and their perceptions of how supportive the environment was at home (r_s_ = −0.48, *p* = 0.005), school (r_s_ = −0.56, *p* = 0.001), and the community (r_s_ = −0.58, *p* = 0.02) meaning that less perceived supportiveness was associated with a higher desire for change in participation.

### 2.4. Statistical Analysis

Statistical analysis was performed using the SPSS 22. Before hypothesis testing, preliminary analyses determined the psychometric properties of the SB-HRQOL and the PEM-CY. Means, standard deviations, and percentages were used to characterize the participants. HRQOL and environmental factors were determined by calculating test scores: two for HRQOL of children and adolescents, and five—for the environment (Table 1) in each setting (home, school, community). Graphical methods (histograms, Q–Q plots) and Shapiro–Wilk’s test were used to check the normality of the data. Kruskal–Wallis test was used to determine differences in the HRQOL between children with the different levels of the SB lesions.

Stepwise multiple linear regression analyses were conducted to test whether the children’s pathology and environmental factors could explain the HRQOL. The regression model examined the HRQOL of children and adolescents separately: thus, two models were tested. Initial examinations of correlations and t-tests revealed that age, gender, home, school, and community environmental supports did not have a significant association with the HRQOL of children and, therefore, were not entered into the model.

Tests with P < 0.05 were considered to be statistically significant. Analysis of histograms, normal P-P plots of the residuals, and scatterplots of the residuals showed that the assumption of homoscedasticity was met. An overall R2 was reported (in percentage) to evaluate the extent to which the examined variables could explain the variance of participation.

Assuming a power of 0.80, and an alpha of 0.05, a sample of 97 is required to detect the medium effect size (Fsq = 0.15) for analyses with six predictors and a single dependent variable [13]. Thus, the current study had enough power to detect medium-to-large effect sizes.

## 3. Results

One hundred and twelve children met the inclusion criteria, and 99 families (88%) agreed to participate in the study. One child with SB and severe acquired brain injury was excluded.

Within the collected sample, there were 60 (61%) children aged from 5 to 12 years (mean ± SD = 8.47 ± 2.28), 32 males and 28 females, and 39 (39%) adolescents aged 13 to 17 years (mean, 14.96 ± 1.78), 20 males and 19 females. The level of spinal cord lesion and related health problems are presented in Table 2. Each child had 1 to 12 comorbidities (mean, 5.33 ± 3.55), and each adolescent had 1 to 10 comorbidities (mean, 6.62 ± 2.7).

The SB-HRQOL scores were moderate for both children and adolescents. The total scores of the SB-HRQOL for children ranged from 73 to 208 out of a possible 220 (mean, 157.08 ± 4.11). The total scores for adolescents ranged from 100 to 217 out of a possible 235 (mean, 158.9 ± 4.32). The children’s SB-HRQOL items mean rating was 3.38 (±0.93) on a scale of 1 to 5, and for adolescents respectively 3.37 (±1.05). Approximately 90% of children and 84% of adolescents in the sample were scored in the positive range (i.e., above 50% on the SB-HRQOL scale). The level of the lesion and the level of ambulation were not associated with the SB-HRQOL both for children and adolescents.

The mean scores of environmental barriers and supports differed considerably across settings. The highest percentage of environmental barriers was found in the community (24%), followed by the preschool/school setting (15%), and home (11%). The highest percentage of environmental supports was found at the preschool/school setting (28%), followed by home (23%), and the community (17%). The PEM-CY summary scores of the environment are provided in Table 3.

The most frequent environmental support for children in all settings was the time that family members spend to promote the participation of a child with SB (35–62%). Access to personal transportation was also a common support for involvement in both preschool/school (44%) and community (49%) settings. The most significant barriers to participation were lack of programs and services to promote the involvement in the community (35%), home (34%), and school or kindergarten (24%). Another common barrier to community participation was the lack of public transportation to access community activities (35%). The physical demands of typical school activities significantly affected participation in school/kindergarten activities (20%).

The most common environmental supports for adolescents were the same as for children: the time that family members spend to promote the participation (21–38% in different settings) and access to personal transportation (38% for participation in the community and 30% at preschool/school settings). Barriers to participation were more prevalent in the community. The most frequent ones were a lack of program and services (38%), physical demands of typical community activities (34%), and inaccessible public transportation (32%).

A multivariate linear regression (Table 4) revealed that the most potent predictors of the HRQOL of children were the number of health conditions, community overall environmental supports, access to personal transportation, and supplies (e.g., assistive devices or technology), explaining 80.3% of the variance in the SB-HRQOL scores. The most potent predictors for adolescents were the number of health conditions, cognitive demands of the activities at home, supplies, physical layout (e.g., presence of sidewalks, availability of ramps or elevators) at school, money, and access to public transportation, accounting for 89.5% of the variance in the SB-HRQOL scores.

## 4. Discussion

In our study of 99 children with SB, we found that both clinical and environmental factors determined the HRQOL of children and adolescents with SB. Environmental supports and resources contributed to HRQOL even more than medical problems. The number of associated medical problems, reflecting disease severity, was the more potent clinical predictor compared to an individual health problem.

An advantage of the study is the nationally represented sample and sufficient statistical power to address the primary research question. An innovative, comprehensive, and psychometrically robust measure was used to capture different aspects of the environment across all settings. However, the study has limitations, which need to be mentioned. The main limitation was the use of parents’ reports to quantify the HRQOL of their children. HRQOL is considered to be a subjective personal experience; therefore, parents’ reports may not fully reflect a child’s perception of the impact of their health condition on daily life. The study did not examine family factors (i.e., general family functioning, income) that also might have influenced HRQOL as well as supports and barriers for children with SB. In terms of the clinical details, we did not include obesity and pressure sores as a potential predictor of the HRQOL which also might have influenced HRQOL. The assessment of the neurological deficit was given in terms of the motor level, not the more precise sensory level, which is a better predictor of functional outcomes.

Previous studies have found inconsistent data regarding a negative impact of pathology on the HRQOL of children with SB. A lot of studies failed to prove the associations between hydrocephalus [14,15,16], bladder and/or bowel incontinence [17,18,19,20,21,22,23,24,25], level of lesion, functional status [16,20,26,27,28] and HRQOL. Interestingly, some studies found even the opposite relationships between pathology and QOL, whereby the higher levels of the lesion and consequently more severe functional limitations were associated with higher levels of QOL [18,21,29,30,31]. These results were interpreted in several ways: (1) healthy adaptation to medical condition which is influenced by protective (e.g., family functioning and environmental supports) factors or resiliency (e.g., hope and locus of control) [17,18,19,23,24,28]; (2) the “marginality hypothesis,” which explains that children with the less severe disability have more challenges with adjustment, i.e., are unable to fit in with their nondisabled peers, but also do not identify themselves with severely disabled children [21]; (3) the imperfection of QOL measures [17,19,22,23]; and (4) profound multisystem disability, which means that the absence of impairment in one system may not be sufficient to improve patient’s overall perceptions of QOL [2,23]. We have tested the latter hypothesis and proved that the HRQOL of children and adolescents with SB depends on the cumulative effect of the impairments in many systems (i.e., severity of the condition) rather than on separate pathological conditions. These findings confirm that children and adolescents with SB need a multidisciplinary team of specialists who can address the various dimensions of medical problems, and an integrated system to deliver this complex care. Also, these findings should encourage practitioners to monitor the outcomes of children with severe SB-related disability most carefully and more readily refer them to mental health and social services.

As expected, children with an increasing number of SB-related comorbidities had poorer HRQOL, which is consistent with some previous research. Cate et al. (2002) assessed children with SB using a parent-reported measure of HRQOL and found that children with more severe impairments had an overall lower HRQOL [14]. Similar findings were reported in the paper of Wang et al. (2013), who used a parent-reported HRQOL measure to explore the associations between the SB-associated medical problems and the HRQOL of children and adolescents with SB [5]. The results of the study showed that the number of associated medical conditions, indicating the severity of illness, was related to both physical and psychosocial health. In contrast, the study of Muller-Godeffroy et al. (2008) investigated self-reported HRQOL of children and adolescents with SB and reported the absence of a linear inverse association between condition severity and HRQOL [15]. The contradictory results of the studies might be due to differences in the age of participants, the methods used to determine the severity of the condition, QOL instruments, and the method of collecting information (parent-reported vs. self-reported HRQOL).

The findings of our study generate knowledge that the HRQOL of children and adolescents with SB is predicted by the existence or lack of environmental barriers and supports. The HRQOL in the study was determined by the variety of environmental characteristics: general environmental factors (e.g., physical layout), the specific demands (e.g., physical and cognitive) of the activity, and availability of resources (i.e., time, transportation, services and programs, money, supplies). The impact of environmental factors on the HRQOL of children with SB has so far been addressed only in a few studies, but only individual ones. Cate et al. (2002) examined the impact of family resources on the QOL of children with SB and hydrocephalus and detected significant associations between the child’s HRQOL scores and family resources [14]. Padua et al. (2002; 2004), assessing the impact of disability on the HRQOL of adolescents with SB, included the presence of an assistant teacher for handicapped in the school as a potential determinant of HRQOL. As expected, the presence of a teaching assistant was associated with a better emotional QOL [30,31]. Another study of Law et al. (2014) explored environmental factors that predict these HRQOL of children with physical disabilities including SB. Environmental barriers had negative and significant associations with psychosocial and physical domains of QOL [32]. Interestingly, the effect of perceived environmental barriers to participation increased with age and was associated mostly with school followed by the physical and structural environment, and policies. In our study, the environmental variables also were the more potent determinant of the HRQOL of adolescents rather than children. These findings are likely to reflect the changes that occur as children reach adolescence and attempt to expand their recreation and leisure activities beyond home and school. With less involvement of parents to mediate environment and low community resources, the perceived influence of environmental barriers and supports increases.

## 5. Conclusions and Implications

Findings from this study indicate the presence of multiple environmental factors affecting the HRQOL of children with SB. Initiatives in the local and national administrative levels (universal design of the physical and structural environment, policy initiatives to increase the availability of personal and public transportation, services, and supplies, etc.) could make a change. In the clinical setting, professionals with an expertise in facilitating activity and participation should assist parents and teachers in adapting already existing, creating new, and implementing effective strategies to engage children into family, school, and community life.

Also, our findings indicate that HRQOL of children and adolescents is strongly affected by the complexity of health condition and thus confirm that a well-organized, regular follow-up by a multidisciplinary team aimed to reduce these various dimensions of medical problems is the best way to deliver healthcare for children and adolescents with SB.

## Figures and Tables

**Table 1:** Descriptions of Participation and Environment Measure for Children and Youth (PEM-CY) summary scores.

Supports	The number of ratings “usually helps” or “usually, yes” divided by the number of items within the setting multiplied by 100. Higher percentages indicate a greater number of supports within a setting.
Barriers	The number of ratings “usually makes harder” or “usually no” divided by the number of items within the setting multiplied by 100. Higher percentages indicate a more significant number of barriers within a setting.
Environmental helpfulness	The sum of the environmental helpfulness ratings within the setting divided by the maximum possible scores of the environment helpfulness within a setting multiplied by 100. Higher scores indicate that the environment is more helpful while supporting the child’s participation in that setting.
Environmental resources	The sum of the environmental resources ratings within the setting divided by the maximum possible scores of the environment resources within a setting multiplied by 100. Higher scores indicate that the resources are more available to support the child’s participation in the setting.
Overall environmental supports	The sum of ratings divided by the number of items rated within the setting multiplied by 100. Higher percentages reflect greater environmental supportiveness.

**Table 2:** Level of the lesion and secondary impairments related to spina bifida.

	Children *n* = 60	Adolescent *n* = 39
Level of lesion		
Thoracic, n (%)	16 (26.7)	3 (7.7)
L1–L2, n (%)	11 (18.3)	8 (20.5)
L3–L5, n (%)	21 (35)	13 (33.3)
Sacral, n (%)	12 (20)	15 (38.5)
Shunting, n (%)	35 (58.3)	24 (66.7)
Number of revisions, mean ± SD (range)	2.94 ± 2.014 (1–12)	3.78 ± 2.082 (1–10)
Arnold Chiari malformation surgery, n (%)	7 (11.7)	3 (7.7)
Surgery for syringomyelia, n (%)	2 (3.3)	0 (0)
Tethered cord surgery, n (%)	1 (1.7)	3 (7.7)
Epilepsy, n (%)	6 (10)	6 (15.4)
Urinary incontinence, n (%)	45 (75)	34 (87.2)
Use incontinence pads or diapers sometimes, n (%)	5 (8.3)	4 (10.3)
Use diapers permanently, n (%)	40 (66.7)	30 (76.9)
Bowel incontinence, n (%)	35 (58.3)	25 (64.1)
Use incontinence pads or diapers sometimes, n (%)	3 (5)	1 (2.6)
Use diapers permanently, n (%)	32 (53.3)	24 (61.5)
Intellectual disability, n (%)	17 (28.2)	18 (46.2)
Mild, n (%)	6 (10)	6 (15.4)
Moderate, n (%)	2 (3.3)	10 (25.6)
Severe, n (%)	2 (3.3)	1 (2.6)
Profound, n (%)	2 (3.3)	1 (2.6)
Severity unspecified, n (%)	5 (8.3%)	0 (0%)
Ambulation		
Normal ambulation, n (%)	18 (30)	8 (20.5)
Community ambulator, n (%)	9 (15)	4 (10.3)
Household ambulator, n (%)	4 (6.7)	6 (15.4)
Nonfunctional ambulator, n (%)	3 (5)	1 (2.6)
Nonambulator, n (%)	26 (43.3)	20 (51.3)
Spinal deformity *, n (%)	28 (46.7)	9 (23.1)
Hip flexion contracture ≥20, n (%)	22 (36.7)	20 (51.3)
Hip dislocation, uni- or bilateral, n (%)	29 (48.3)	9 (23.1)
Knee flexion contracture ≥20, n (%)	24 (45)	19 (48.7)
Foot deformities, n (%)	34 (56.7)	27 (69.2)
Low-impact fractures, n (%)	14 (23.3)	13 (33.3)

* Defined as scoliosis with Cobb angle ≥ 30 and/or major sagittal deformity (either lordosis or kyphosis of 70° or more) or previously surgically treated spine deformity with no curvature at present examination

**Table 3:** Summary scores of environmental scales for each setting.

Setting	Scale	Summary Scores			
		Mean	Standard Deviation	Minimum	Maximum
Home	Supports (%)	22.94	15.65	0	75
	Barriers (%)	11.38	12.11	0	42
	Environmental helpfulness (%)	92.37	8.15	70	100
	Environmental resources (%)	73.39	14.14	40	100
	Overall environmental supports (%)	84.73	8.51	64	100
Preschool/school *	Supports (%)	28.72	13.52	6	71
	Barriers (%)	14.52	12.22	0	53
	Environmental helpfulness (%)	84.45	10.85	52	100
	Environmental resources (%)	78.01	12.31	50	100
	Overall environmental supports (%)	81.35	9.29	59	100
Community	Supports (%)	17.24	10.8	0	50
	Barriers (%)	23.92	17.34	0	63
	Environmental helpfulness (%)	75.59	12.92	48	100
	Environmental resources (%)	68.76	14.09	38	100
	Overall environmental supports (%)	72.66	11.52	50	100

* Data on preschool/school participation were calculated for 85 participants who were attending school or kindergarten.

**Table 4:** Statistical results from the regression models.

Model	B	SE B	β	R2 (%)	95% CI for B	t	p
*SB-HRQOL of children*							
Community overall environmental supports	1.059	0.228	0.504	80.3	(0.6–1.518)	4.642	0.0001
Number of health conditions	−2.875	0.708	−0.395		(−4.3 to −1.45)	−4.062	0.0001
Access to personal transportation	5.917	2.04	0.236		(1.805–10.029)	2.9	0.023
Supplies at preschool/school	6.928	3.118	0.181		(0.653–13.204)	2.222	0.031
*SB-HRQOL of adolescents*							
Number of health conditions	−4.05	0.85	−0.387	89.5	(−5.794 to −2.307)	−4.766	0.0001
Cognitive demands of activities at home	14.143	2.835	0.345		(8.327–19.96)	4.989	0.0001
Supplies	12.637	3.266	0.267		(5.935–19.339)	3.89	0.0001
Money	10.855	2.427	0.303		(5.876–15.834)	4.473	0.0001
Physical layout at school	9.541	3.305	0.188		(2.761–16.332)	2.887	0.008
Access to public transportation	6.973	2.802	0.206		(1.224–12.723)	2.489	0.019

SB-HRQOL, Spina Bifida Health-Related Quality of Life.
